# Data on a highly stable electrocatalyst of NiCoPt/Graphene-dot nanosponge for efficient hydrogen evolution reaction

**DOI:** 10.1016/j.dib.2020.106332

**Published:** 2020-09-24

**Authors:** Ngoc-Anh Nguyen, Yousuf Ali, Van-Toan Nguyen, Oleksii Omelianovych, Liudmila L. Larina, Ho-Suk Choi

**Affiliations:** a Department of Chemical Engineering and Applied Chemistry, College of Engineering, Chungnam National University, 99 Daehak-ro, Yuseong-Gu, Daejeon, 34134, Republic of Korea

**Keywords:** NiCoPt/Graphene-dot, Nanosponges, Electrocatalysts, Hydrogen evolution reaction

## Abstract

The data presented in this article are related to the research article entitled “NiCoPt/Graphene-dot Nanosponge as a Highly Stable Electrocatalyst for Efficient Hydrogen Evolution Reaction in Acidic Electrolyte (N.-A. Nguyen et al., 2020) [1]. This article reports a simple method to synthesize NiCoPt/Graphene-dot as an electrocatalyst with low Pt loading but high hydrogen evolution reaction (HER) performance. The morphology of NiCoPt/Graphene-dot was analyzed by scanning electron microscopy (SEM) and high-resolution transmission electron microscopy (HRTEM) techniques. The structural and chemical properties of NiCoPt/Graphene-dot were investigated by using X-ray Powder Diffraction (XRD) and X-ray photoelectron spectroscopy (XPS) techniques.

## Specifications Table

**Table:** 

Subject	Physics, Chemistry
Specific subject area	Electrochemical catalysts for hydrogen evolution reaction
Type of data	Table Image Graph Figure
How data were acquired	Evaluation of the characterizations of synthesized electrocatalyst: The structure of obtained samples was investigated by using Powder X-ray diffraction (XRD) measurement with a Cu target (Cu Kα1 = 1.541 Å), (Japan). The measurements were conducted from 15° to 60° with steps of 0.02°. The morphology was observed by scanning electron microscopy (FE-SEM, Hitachi S-4800 with UHR lens) and by high-resolution transmission electron microscopy (HRTEM, JEM-2100F, 200 kV, JEOL LTD., Japan). X-ray photoelectron spectroscopy (XPS) characterization was performed on a Thermo Fisher Theta Probe system equipped with a monochromated Al-K X-ray source with a photon energy of 1486.6 eV. (K-Alpha+, Thermo Fisher Scientific).
Data format	Raw Analyzed Filtered
Parameters for data collection	Data on a synthesis of NiCoPt/Graphene-dot Nanosponge electrocatalyst including its characteristics and electrochemical properties.
Description of data collection	The samples were synthesized by the co-reduction method, then their characteristics were analyzed by XRD, SEM, TEM, and XPS methods. The electrochemical performance was collected by a potentiostat (IViumStat)
Data source location	Chungnam National University City: Daejeon Country: Republic of Korea
Data accessibility	With the article
Related research article	Ngoc-Anh Nguyen, Yousuf Ali, Van-Toan Nguyen, Oleksii Omelianovych, Liudmila L. Larina, Ho-Suk Choi*, “NiCoPt/Graphene-dot Nanosponge as a Highly Stable Electrocatalyst for Efficient Hydrogen Evolution Reaction in Acidic Electrolyte.” Journal of Alloys and Compounds doi:10.1016/j.jallcom.2020.156651

## Value of the Data

• This data provides the scientific community in the electrocatalysis field a simple method to synthesize Ni_48_Co_48_Pt_4_/Graphene-dot (Ni_48_Co_48_Pt_4_/G-dot) as an efficient electrocatalyst in the application of hydrogen evolution reaction.
• SEM and TEM images are taken to see the particle morphology and size of Ni_48_Co_48_Pt_4_/Graphene-dot, from which the scientists could predict the electrocatalytic performance of catalysts.
• XRD pattern suggests that Ni_48_Co_48_Pt_4_/G-dot possesses a carbon amount on the surface of nanoparticles, which can explain for the improvement of the stability of catalyst in the water-splitting process.
• Advanced XPS technique with the etching process of the sample before XPS measuring is examined to understand the electronic structure of electrocatalyst to explain for scientists how the activity of the catalyst can be enhanced.
• Electrochemical tests on Ni_48_Co_48_Pt_4_/Graphene-dot and Ni_48_Co_48_Pt_4_ nanoalloy are performed to see the improvement of hydrogen evaluation reaction (HER) performance of Ni_48_Co_48_Pt_4_/Graphene-dot.

## 1 Data Description

**Fig. 1:**
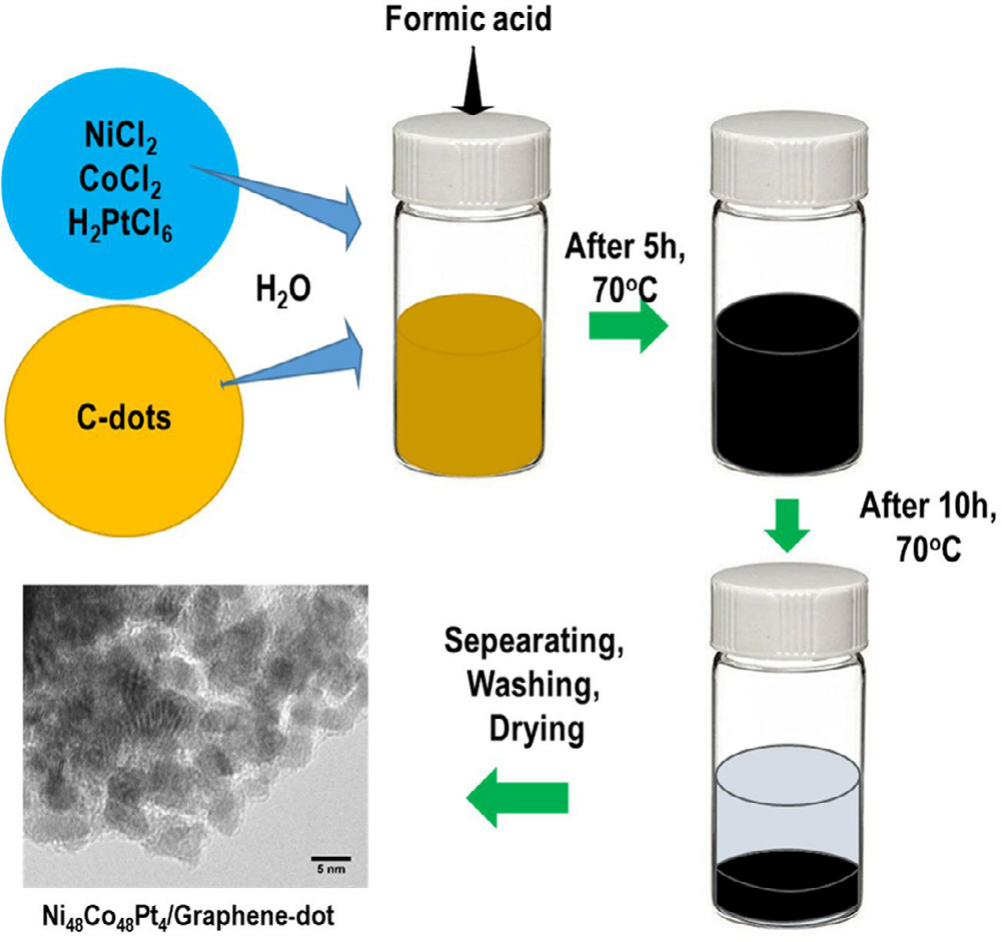
Schematic representation of the synthesis of NiCoPt/G-dot nanosponge.

**Fig. 2:**
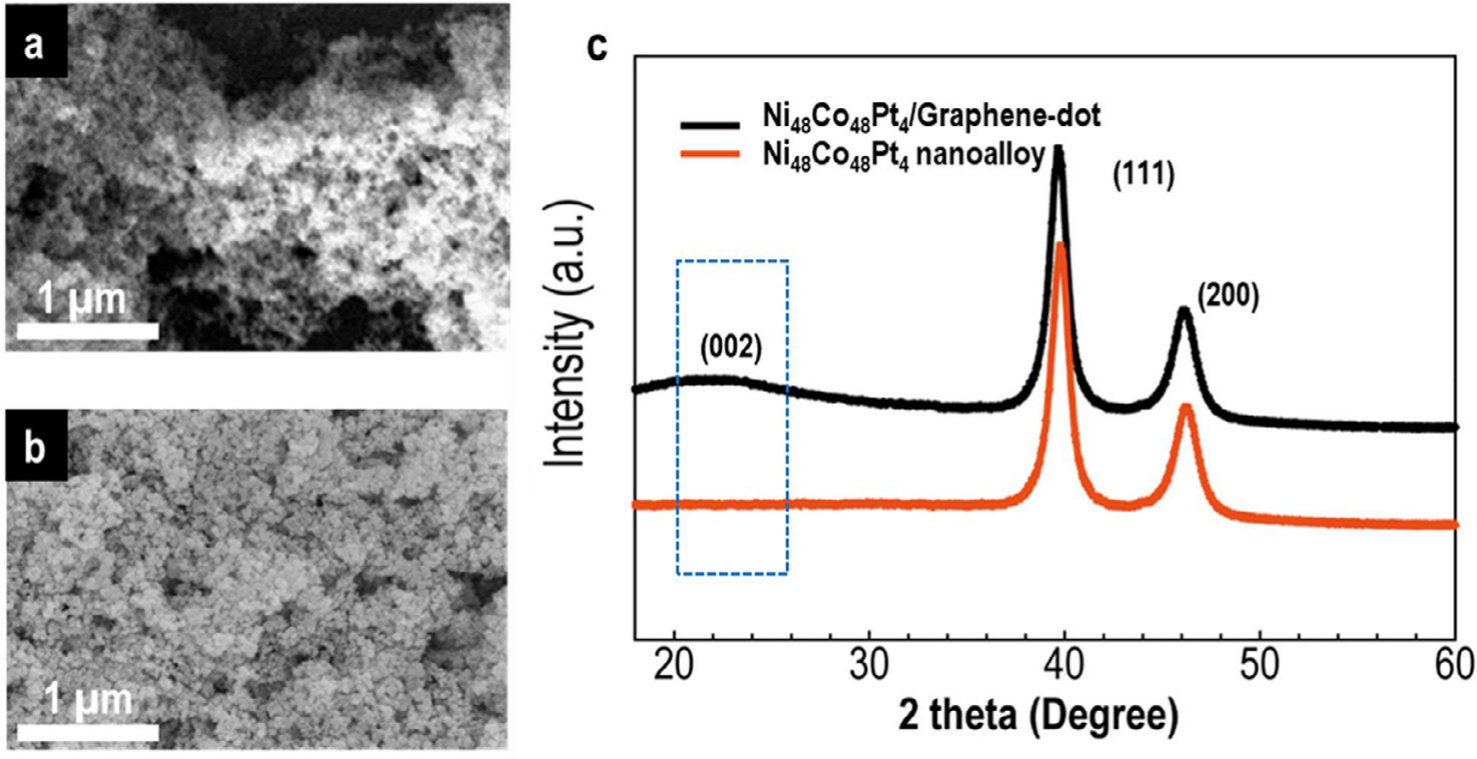
(a) and (b) SEM images of Ni_48_Co_48_Pt_4_/G-dot and Ni_48_Co_48_Pt_4_ nanoalloy, respectively. (c) X-ray diffraction patterns of Ni_48_Co_48_Pt_4_/G-dot and Ni_48_Co_48_Pt_4_ nanoalloy. The broad peaks at 24.35˚ indicates the formation of graphene in Ni_48_Co_48_Pt_4_/G-dot.

**Fig. 3:**
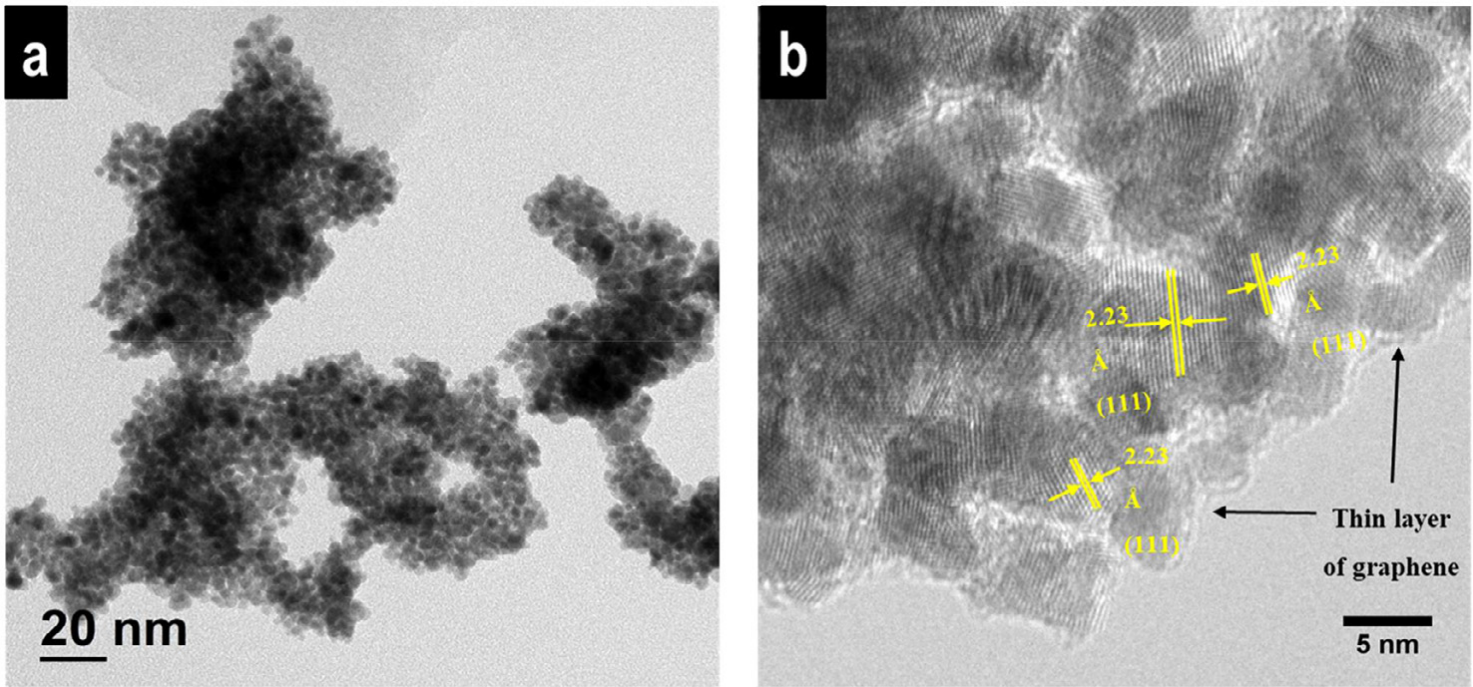
(a) TEM and (b) HRTEM images of Ni_48_Co_48_Pt_4_/G-dot nanosponge.

**Fig. 4:**
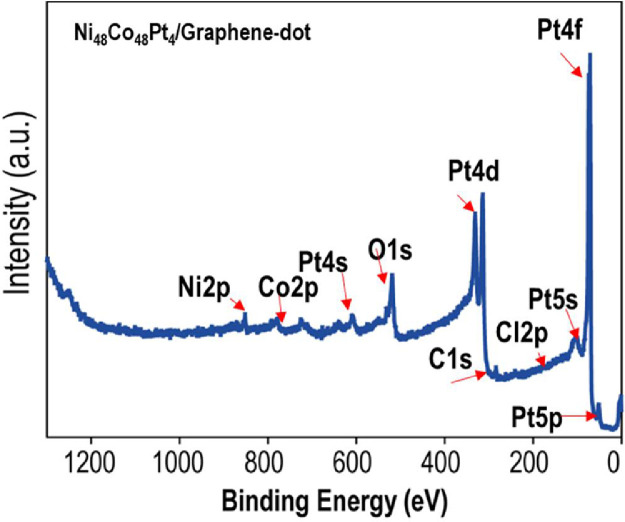
The survey XPS spectrum was taken from Ni_48_Co_48_Pt_4_/G-dot nanosponge after a mild etching of the sample with the acceleration energy of 0.5 keV.

The data of this article provides information on the synthesis of Ni_48_Co_48_Pt_4_ alloy wrapped with graphene dots, which shows the high HER performance as well as very stable in the long-term of hydrogen production. Fig. 1 gives a synthesis process of Ni_48_Co_48_Pt_4_/Graphene-dot (mentioned as Ni_48_Co_48_Pt_4_/G-dot). Fig. 2 presents the morphology and structure of Ni_48_Co_48_Pt_4_/G-dot and Ni_48_Co_48_Pt_4_ nanoalloy. Fig. 3 shows the morphology and lattice fringes with inter-planner distances of 2.23 Å corresponding to the (111) crystal plane. Fig. 4 supplies the survey XPS spectrum to disclose the electronic structure of Ni_48_Co_48_Pt_4_/G-dot. Table 1 indicates the detailed values of electrochemical performance in the hydrogen evolution reaction (HER) application.

**Table 1:** Electrochemical performance data of Ni_48_Co_48_Pt_4_/G-dot, Ni_48_Co_48_Pt_4_ nanoalloy, and commercial Pt/C catalysts.

Electrocatalysts	Overpotential (mV)	Tafel slope (mV.dec^−1^)	R_ct_ (Ω)	C_dl_ (mF)	ECSA (cm^2^)
Ni_48_Co_48_Pt_4_ nanoalloy	52.70	37.62	47.15	1.599	45.71
Ni_48_Co_48_Pt_4_/G-dot	45.54	33.90	29.05	2.013	57.51
Pt/C	41.60	30.23	19.60	2.080	59.43

After synthesizing Ni_48_Co_48_Pt_4_/G-dot sample as described in Figure 1, the morphology and structure of obtained catalysts were investigated as seen in Fig. 2. Clearly, in the presence of C-dots in the synthesis process, the morphology of Ni_48_Co_48_Pt_4_/G-dot is sponge-like in contrast to Ni_48_Co_48_Pt_4_ nanoalloy (synthesized without C-dots) with nanoparticles that tend to be aggregated (Fig. 2a, b). Fig. 2c shows the XRD patterns of Ni_48_Co_48_Pt_4_/G-dot and Ni_48_Co_48_Pt_4_ nanoalloy samples. In detail, the pattern of Ni_48_Co_48_Pt_4_/G-dot nanosponge, a weak and broad peak at approximately 24.35˚ representing the crystal plane of (002) is observed. However, no peak is found at 24.35˚ in the pattern of Ni_48_Co_48_Pt_4_ nanoalloy. The obtained result indicates the formation of graphene layers in Ni_48_Co_48_Pt_4_/G-dot, which wraps the synthesized catalyst and creates a sponge-like polycrystalline structure [2]. The morphology of Ni_48_Co_48_Pt_4_/G-dot nanosponge is confirmed again by TEM as seen in Fig. 3a. The nanoparticles wrapped by graphene layers are clearly seen in Fig. 3b. Fig. 4 shows the survey XPS spectrum of Ni_48_Co_48_Pt_4_/G-dot nanosponge, which was treated under a mild acceleration Ar^+^ energy of 0.5 keV. The high-resolution of Ni2p, Co 2p, Pt4f, and C1s spectra are deconvoluted as seen in the reference [1]. As a result, the carbon amount has recorded of 25.43 at%, suggesting that the surface of the nanoparticle is enriched with carbon. On the other hand, atomic percentages of metal elements obtained from survey spectra taken from Ni_48_Co_48_Pt_4_/G-dot nanosponge are given with 86.69 % of atomic percentage for Pt while only 7.14 and 6.17% are found for Co and Ni. The top layer is enriched up to 86.69% Pt suggesting that the surface composition of an alloy is controlled by the tendency of Pt metal segregates towards the surface. This result can be used to explain why the HER performance of Ni_48_Co_48_Pt_4_/G-dot nanosponge is excellent as seen in Table 1.

The beneficial effect of graphene-dot wrapped nanosponge on the HER activity is proven by direct comparison of the HER performance of the Ni_48_Co_48_Pt_4_ with and without G-dots as seen in Table 1. The overpotential value of 52.70 mV for Ni_48_Co_48_Pt_4_ nanoalloy is higher than that of Ni_48_Co_48_Pt_4_/G-dot (45.54 mV) to obtain a current density of 10 mA.cm^−2^. The obtained data prove that the coverage of the nanoparticles with G-dots not only enhances durability but also increases its electrical conductivity and provides a favorable catalyst/electrolyte interface for electron transfer from the electrode to the protons in the electrolyte.

The beneficial impact of the G-dot is further confirmed by the EIS, Tafel plots, and the double layer capacitance analysis. The Tafel slope of Ni_48_Co_48_Pt_4_/G-dot is 33.90 (mV/dec), which is smaller than the slope of 37.62 mV/dec recorded for Ni_48_Co_48_Pt_4_ nanoalloy. This data suggests that the electrochemical recombination step is the rate-determining step and the reaction follows the Volmer-Tafel mechanism [3], [4], [5]. The ECSA of Ni_48_Co_48_Pt_4_/G-dot is 57.51 cm^2^, which is larger than that of 45.71 cm^2^ of Ni_48_Co_48_Pt_4_ nanoalloy. In addition, the smaller value of 29.05 Ω for R_ct_ is fitted for the Ni_48_Co_48_Pt_4_/G-dot compared to the R_ct_ of 47.15 Ω recorded for Ni_48_Co_48_Pt_4_ nanoalloy, suggesting a more effective charge transfer across the catalyst/electrolyte interface that promotes the electrochemical reaction.

The comparative chronoamperometric curves recorded for Ni_48_Co_48_Pt_4_ alloy and Ni_48_Co_48_Pt_4_/G-dot are given in the reference [1]. The 40% loss of current density after 18 h of operation was recorded for Ni_48_Co_48_Pt_4_ nanoalloy, whereas Ni_48_Co_48_Pt_4_/G-dot nanosponge retains 94% of the current density after 21 h, showing an excellent catalytic activity. The result illustrates the positive impact of the G-dot in the stability of Ni_48_Co_48_Pt_4_/G-dot catalyst in acidic electrolyte.

## 2 Experimental Design, Materials, and Methods

### 2.1 Materials

Nickel chloride hexahydrate (NiCl_2_•6H_2_O, 99.99%), cobalt chloride hexahydrate (CoCl_2_•6H_2_O, 99.9%), chloroplatinic acid hexahydrate (H_2_PtCl_6_•6H_2_O, 99.9%), formic acid (HCOOH, 95 %, reagent grade), and ethanol (C_2_H_5_OH, 99.99%) were obtained from Sigma-Aldrich, USA. The Nafion D521 solution (5 wt%) was bought from Dupont (USA).

### 2.2 Methods

NiCoPt/Graphene-dots (referred to as NiCoPt/G-dot) were synthesized from the mixed precursor solutions and carbon dots (C-dots). C-dots were synthesized using a procedure developed in our previous work [2,6,7]. A typical synthesis for the Ni_48_Co_48_Pt_4_/G-dot nanohybrid can be described as seen in Fig. 1. Ni_48_Co_48_Pt_4_ nanoalloy was synthesized with the same method to obtain NiCoPt/G-dot except using C-dots.

### 2.3 Experimental design

After synthesizing Ni_48_Co_48_Pt_4_/G-dot and Ni_48_Co_48_Pt_4_ nanoalloy samples, their physical characteristics such as crystalline structure, morphology, and surface chemical state were analyzed by using techniques such as the Powder X-ray diffraction (XRD), the scanning electron microscopy (SEM) and transmission electron microscopy (TEM), and X-ray photoelectron spectroscopy (XPS), respectively. In more detail, an X-ray diffractometer (Smart Lab, λ = 1.5406 Å, Cu Kα radiation, Rigaku corporation) was used to analyze the crystalline structure of the NiCoPt/G-dot nanosponge. Scanning electron microscopy (FE-SEM, Hitachi S-4800 with UHR lens) and high-resolution transmission electron microscopy (HRTEM, JEM-2100 F, 200 kV, JEOL LTD., Japan) were used to analyze the morphology of the synthesized NiCoPt/G-dot nanosponge. X-ray photoelectron spectroscopy (XPS) using a Thermo Fisher Theta Probe system equipped with a monochromated Al-K X-ray source with a photon energy of 1486.6 eV (K-Alpha+, Thermo Fisher Scientific) was used to analyze the surface chemical state of synthesized sample.

For measuring the electrochemical performance of the NiCoPt/G-dot catalyst, a three-electrode scheme with a rotating disc electrode was employed, using a potentiostat (IviumStat electrochemical analyzer from Ivium Technologies, Netherlands). NiCoPt/G-dot catalyst coated on glassy carbon (GC) electrode was used as the working electrode (WE). A platinum coil and a Ag/AgCl (NaCl 3 M) electrode were used as the counter and reference electrodes, respectively. The electrochemical catalytic activity of the NiCoPt/G-dot was performed in the acidic electrolyte (0.5 M H_2_SO_4_) by linear sweep voltammetry (LSV) at a scan rate of 10 mV/s. The electrolyte was degassed by bubbling with ultra-pure nitrogen gas for 30 min before the measurements. The electrochemical impedance spectroscopy (EIS) was examined at a voltage of -0.20 V vs a reversible hydrogen electrode (RHE) in a frequency range of 0.1 to 10^5^ Hz.

## Ethics Statement

The data resulted from experimental neither on animal models nor with human volunteers.

## Declaration of Competing Interest

The authors declare that they have no known competing financial interests or personal relationships which have, or could be perceived to have, influenced the work reported in this article.

## Appendix Supplementary materials

Image, application 1

Image, application 2

Image, application 3

Image, application 4

Image, application 5

Image, application 6

Image, application 7

Image, application 8

Image, application 9

## References

[1] Nguyen N.-A., Ali Y., Nguyen V.-T., Omelianovych Oleksii, Larina Liudmila L., Choi H.-S. (2020). NiCoPt/Graphene-dot Nanosponge as a Highly Stable Electrocatalyst for Efficient Hydrogen Evolution Reaction in Acidic Electrolyte. J. Alloys Compd..

[2] Nguyen V.-T., Nguyen N.-A., Ali Y., Tran Q.C., Choi H.-S. (2019). Graphene dot armored PtMo nanosponge as a highly efficient and stable electrocatalyst for hydrogen evolution reactions in both acidic and alkaline media. Carbon.

[3] Nguyen N.-A., Nguyen T.T.-T., Nguyen V.-T., Ali Y., Sim E., Choi H.-S. (2019). Plasma-treated sponge-like NiAu nanoalloy for enhancing electrocatalytic performance in hydrogen evolution reaction. Catal. Today.

[4] Nguyen N.-A., Nguyen V.-T., Shin S., Choi H.-S. (2019). NiRh nanosponges with highly efficient electrocatalytic performance for hydrogen evolution reaction. J. Alloys Compd..

[5] Cao X., Han Y., Gao C., Xu Y., Huang X., Willander M., Wang N. (2014). Highly catalytic active PtNiCu nanochains for hydrogen evolution reaction. Nano Energy.

[6] Nguyen V.-T., Tran Q.C., Quang N.D., Nguyen N.-A., Bui V.-T., Dao V.-D., Choi H.-S. (2018). N-doped Cdot/PtPd nanonetwork hybrid materials as highly efficient electrocatalysts for methanol oxidation and formic acid oxidation reactions. J. Alloys Compd..

[7] Nguyen V.-T., Ha H., Nguyen N.-A., An H., Kim H.Y., Choi H.-S. (2020). In situ engineering of Pd nanosponge armored with graphene dots using Br–toward high-performance and stable electrocatalyst for the hydrogen evolution reaction. ACS Appl. Mater. Interfaces.

